# A descriptive prospective study of sports medicine practices for athletes in Uganda

**DOI:** 10.4314/ahs.v21i2.43

**Published:** 2021-06

**Authors:** Samuel K Lubega, Timothy Makubuya, Haruna Muwonge, Mike Lambert

**Affiliations:** 1 Exercise Science, Division of Exercise Science and Sports Medicine (ESSM). Department of Human Biology, University of Cape Town, Cape Town, South Africa, 7700; 2 Center for Sports Research in Uganda, Department of Educator Preparation and Leadership, College of Education, University of Missouri- St. Louis, 364 Marillac Hall, 1 University Blvd, St. Louis, MO 63121, USA; 3 Department of Physiology, College of Health Sciences, Makerere University P.O. Box 37580, Kampala (Uganda)

**Keywords:** Injuries, medical, Uganda, emergency, intermediate, rehabilitation, return-to-sports

## Abstract

**Background:**

Many international sporting organizations have recommended practices to reduce the risk of injury. These practices include screening for injury, having appropriate emergency medical care, and protocols for managing injury before return-to-play. The extent of the uptake of these practices in a developing country such as Uganda, is unknown.

**Methodology:**

Using a descriptive case study approach, this investigation focused on a sample of injured athletes (n = 75) in Uganda from four main sports associations (football, athletics, basketball and rugby). The data were collected through observations and interviews after the injury. Using a best medical practice framework the phases of emergency, intermediate, rehabilitative, and return-to-sports participation were described.

**Result:**

Nine conditions/types of injury were included. The results revealed a lack of specific pre-season screening or return-to-play readiness for all the injured athletes. Further, there was a lack of application of best practice principles for most of the injury types. For athletes who received medical care, the results show inconsistencies and inadequacies from the acute stage of the injury to return-to-sports participation.

**Conclusion:**

This study identified barriers such as up-to-date knowledge among the sports resource providers; the gaps for appropriate and adequate specific facilities for managing injured athletes, and policies to mandate care of injured athletes. These barriers detract from applying best medical practices.

## Introduction

As competitive sports have become increasingly popular, there is an increase in the prevalence and incidence of sports related injuries at all levels of participation.^1^–^4^ This can be explained by overuse injuries from high volume of training, rule violations, and unavoidable injuries from contact or collisions between players. 1, 5–7 These consequences not only affect athletes, but the participation in sports at various levels. Injuries impact the health of athletes, have financial consequences and might result in the early retirement of the athletes.^8^ More so, a previous study of Ugandan elite sports participants, revealed that the majority of athletes at secondary and tertiary education levels also participate in sport at elite clubs level,^9^ which predisposes them to injury at an early stage.

Recent epidemiological evidence also suggests that immediate deaths due to cardiac arrest during sports participation, as well as the long-term consequences of disability due to brain damage are major public health concerns.^2^, ^10^–^12^ This evidence is coupled with a current global push for injury prevention and management, 13,14 and documentation of injury occurrences.^15^–^19^ We hypothesize that Uganda is in early stages of sports injury surveillance. The aim of this study was to examine the sports medicine practices in managing athletes before and after injury in Uganda, using the various stages of the best medical practice framework model. The aim of this study was to examine the sports medicine practices in managing athletes before and after injury in Uganda, using the various stages of the best medical practice framework model as a source of comparison.

## Methods

### Study Design and Setting

A descriptive prospective cohort study design was used to document injury prevention and management behaviours. Data on the management of sports injuries were collected in a six-month period through the various phases from emergency to return-to-sports phase using a previously published model.^20^ Athletes, coaches, managers and health care providers were informed and consent was obtained for the observation and followup of their athletes during training and competition for a 6-month study period at various athletic training locations in Uganda. During the study period, seventy-five time-loss injuries were observed and recorded by the primary investigator on a data capture form. The data capture form utilized in this study consisted of five separate sections on demographics, preventive care, emergency care, intermediate care and return-to-sports. The immediate injury data contained demographic information and emergency injury management information that were documented by the researchers through observation and interview. Injury management during the emergency phase of an injury is determined using the five criteria; response to injury on field, immediate field assessment; appropriate removal of an athlete from the field of play/court, side-line (near the court or field) treatment and evaluation.^20^

### Participants selection and recruitment

The study participants were top level national athletes that were selected using purposive sampling from the four of the 48 currently registered sports associations (athletics, basketball, football/soccer and rugby) by Uganda National Council for Sports. Study participants accepted daily calls for the first 15 days following the injury, and then a weekly call thereafter, until a time when they returned to sports participation. Only participants who were identified as belonging to the four sports associations, and who were 18 years of age and above, were included in the study. However, in order to avoid bias in selection all injured athletes that actively participate in activities of the four sports associations during the study period were observed. We excluded underage athletes and a few assistant coaches who occasioally played or participated in practice drills with the participants. Overall, this study considered both female and male participants, who played four different sports, that require different duration of play. These participants had varying levels of injury history and pre-participation screening. The sports medicine practices that are detailed in our study are fundamental in determining the overall health and wellbeing of these athletic participants. This study was approved by the Institutional Review Board (IRB) at University of Cape Town (HRECREF: 584/2014) and the Uganda National Council of Science and Technology (SS3626).

## Results

A summary of the participant's demographic data (average age, gender, injury frequency), and the time-loss injuries recorded from each sporting association are reported in Table 1.

**Table 1:** Demographic Characteristics of Injured Athletes

Sports Associations	Age (years)	Time-loss injuries
Athletics	22.8 ± 2.5	16 (F=6; M=10)
Rugby	23.1 ± 3.0	23 (F=2; M=21)
Basketball	27.8 ± 4.4	25 (F=10; M=15)
Football/soccer	21.7 ± 3.1	11 (M=11)

**Average Total**	23.9±3.3	75 (F = 18; M = 57)

Age expressed as mean ± standard deviation; F = female, M = male

### The emergency phase

Table 2 shows the athletes who fainted did receive emergency care, except for the athlete who fainted, and a few other athletes who had ankle and wrist sprains (8%) and strains (21%) (Table 2). In this case, the primary health care provider was the sports physician, who was assisted by team coaches. Table 2 shows no athletes received side-line services (61%). However, no injury details were recorded. In addition, many of the athletes were never referred for further medical evaluation.

**Table 2:** Services provided to athletes during the emergency phase (on-field care)

Injury or conditions	Emergency care phase		Response to injury		Removal from field		Side-line service		Injury recording		Referred conditions	
	Yes	No	Yes	No	Yes	No	Yes	No	Yes	No	Yes	No
Abrasion	1	-	-	1	-	1	-	1	-	1	-	1
Concussions	3	-	-	3	1	2	-	3	-	3	2	1
Contusion	1	-	-	1	-	1	-	1	-	1	-	1
Dislocations	1	-	-	1	-	1	-	1	-	1	1	-
Lacerations	4	-	-	4	4	-	-	4	-	4	1	3
Fainting	1	-	1	-	-	1	-	1	-	1	1	-
Fractures	3	-	-	3	3	-	-	3	-	3	1	2
Sprain	26	15	3	28	26	5	-	41	-	41	6	36
Strain	12	7	4	15	10	9	-	19	-	19	3	6

### Intermediate phase

At this stage, injured athletes receive further assessments and treatment beyond the emergency phase. The duration of this phase varied and ranged from 5 weeks to 12 weeks, depending on the type of injury. In evaluating the intermediate phase of medical care, the results revealed that only 61% of the injured athletes sought further health care from a medical facility after they had sustained injuries. On reaching the health care facilities, these athletes reported that they were managed through a conservative approach, that is non-invasive and without surgical procedures. These athletes also consulted a physiotherapist, who may not have been trained in sports physiotherapy.

Table 5 illustrates both investigative and treatment modalities utilized to address the injury situations of the referred athletes. The investigative modalities presented to athletes at various medical centres included the computerized tomography (CT)-scan, magnetic resonance imaging (MRI), physical assessments, diagnostic ultra-sound and x-rays. Whereas the treatment modalities available to treat athlete's injuries at this stage included, casting materials, medication, orthopaedic manipulations, ice-therapy and wound dressing materials. Table 5, further shows that the majority of the athletes were treated without qualifying them for further investigations. The concussed athlete at this stage was advised to continue to rest, while the athlete with a fracture injury received medications.

**Table 5:** Investigations and treatment given to athletes with different injuries during the intermediate phase

Health facility services	Abrasion	Concussion	Contusion	Dislocation	Faint	Fractures	Lacerations	Sprain	Strain
CT Scan	-	-	-	-	-	-	-	-	1
Diagnostic U/S	-	-	-	-	-	-	-	3	2
U/S Scan & Physical assessment	-	-	-	-	-	-	-	2	-
Physical assessment	-	-	-	-	-	-	-	3	1
Wound management	-	-	-	-	-	-	1	-	-
Diagnostic MRI	-	-	-	-	-	-	-	1	-
Casting (Plaster of Paris)	-	-	-	-	-	1	-	-	-
Medications (Antibiotics, anti-fungal & Analgesics)	-	-	-	-	-	1	1	1	-
Diagnostic MRI & Scan	-	-	-	-	-	-	-	1	-
Diagnostic MRI & X-ray	-	-	-	-	-	-	-	-	1
Correction shoulder dislocation	-	-	-	1	-	-	-	-	-
Advised to rest	-	1	-	-	-	-	-	-	-
Ice-therapy	-	-	-	-	-	-	-	5	3
Heat-therapy	-	-	-	-	-	-	-	4	-
Diagnostic X-rays	-	-	-	-	-	1	-	3	2
Massage therapy	-	-	-	-	-	-	-	6	2
No treatment	2	2	1	-	1	-	2	12	7

Total	2	3	1	1	1	3	4	41	19

### Rehabilitation phase

Generally, our results indicate that specialists were not utilized by athletes that had sustained sports-related injuries during the rehabilitation stage. Except for athletes who had ankle or wrist sprain or strain injuries, none of the other injured athletes were observed by the key health specialists during the rehabilitation stage of injury management.

### Return to sports participation phase

Table 6 reveals that majority of the athletes who were recovering from sports-related injuries made individual/personal, non-medical decisions to return-to -sports participation. All the athletes who were recovering from abrasions, contusions, dislocation, and loss of consciousness (fainting) returned to sports without clearance by a qualified medical personnel. One-third of athletes who were recovering from concussions and fractures also returned to sports without clearance of a qualified medical personnel.

**Table 6:** Source of permission to return-to-sports (n = 75 injuries)

Clearance	Abrasion	Concussion	Contusion	Dislocation	Faint	Fractures	Lacerations	Sprain	Strain
Coach	-	-	-	-	-	1	-	1	1
Self	2	1	1	1	1	1	2	25	10
Nurse	-	-	-		-	-	1	-	-
Physiotherapist	-	-	-	-	-	-	-	10	2
Doctor	-	2	-	-	-	-	-	-	4
Not yet	-	-	-	-	-	1	-	5	2

Total	2	3	1	1	1	3	3	41	19

## Discussion

### Main Findings and Interpretation

Our study precedes any conducted on Ugandan athletes' sports medicine practices, and is pivotal to alleviate any barriers to the appropriate use of best sports medicine practices. Previous studies have highlighted the need for pre-participation athlete evaluation, as a safety remedy.^21^–^24^ Our study revealed that only 3% of the time-loss injuries occured in athletes that had some form of pre-participation evaluation. The lack of mandatory pre-participation medical evaluation of athletes in Uganda, 25 and documentation of athlete's injury circumstances suggests that these strategies are not common practices among the sports communities in Uganda, thus warranting practitioner and athlete sensitization. Injury management during the emergency phase of an injury is determined using the five criteria; response to injury on field, immediate field assessment; appropriate removal of an athlete from the field of play/court, side-line (near the court or field), treatment and evaluation.^20^ Furthermore, best medical practices involving the documentation of the injury of an athlete is vital, particularly through an on-site facility.

As previously evidenced, intrinsic factors such as the athletes' health status, are a major risk for the various sports related injuries.^7^, ^26^ Furthermore, the lack of pre-participation evaluation of an athlete when returning to sports participation from a previous injury predisposes the athlete to further serious injury risk.^7^ An existing argument suggests that inappropriate and inadequate management of the athlete's musculoskeletal problems causes recurrent symptoms of pain and subsequent injuries.^27^ Likewise, if a first concussion injury is not fully rehabilitated, an increase in the risk of a subsequent injury is possible.^2^, ^7^, ^28^–^31^ Moreover, a recent study on the perception of concussion injuries among Ugandan athletes and coaches, revealed that there are still gaps to be bridged in relation to concussion awareness training in sports.^32^ Results of our current study highlight the risk of adverse injury, among professional Ugandan athletes that participate in sports programs, that are not well equipped with sports health care providers. More so, athlete injury management during the emergency phase was inadequate across all the nine injury conditions. For example, nearly half of the athletes did not receive side-line treatment, yet this is vital for all sports-related injury conditions.^33^, ^34^ Furthermore, findings of our study reveal the inadequacies in medical services during the emergency phase of sports-related injuries among Ugandan athletes. Such inadequacies, may suggest knowledge gaps on emergency management of sports-related injuries amongst service providers,^35^ and the lack of adequately trained or skilled medical staff in sports injuries 36, 37 to effect adherence to vital practices in sports. Largely, the inadequacy in the conduct of investigation and treatment services was demonstrated at intermediate phase of care. For example, about two-thirds of concussed athletes in our study did not report any further evaluation of their injury in the rehabilitation and return-to-sports phase of concussion injury management. It is plausible that the medical providers deemed such injury as non-life threatening. Besides, the advice to rest reported by the injured athlete at this stage without further monitoring, deviates from that recommended in the Concussion in Sport Group Consensus Statement. 37 However, it is important to recognize that some low and middle income countries, such as Kenya and Ethiopia have already begun to initiate stringent protocols and challenging concussions early enough.^38^,^39^ In Uganda, efforts are underway to equip primary school and secondary school youth, sports teachers and coaches with relevant sports injury prevention strategies through their sports curriculum.^40^ This is a vital step for the preparation of both amateur and professional athletes in Uganda.

Although MRI, Ultra-sound or CT scan modalities have been suggested to be the most efficient investigative modalities for musculoskeletal injuries, 41 the fracture in the current study reported undergoing such investigation. The findings of the current study may suggest the lack of resourced medical facilities to manage athlete injuries in Uganda. Further research should be directed towards assessing medical facilities on the availability of personnel and equipment required to prevent and manage common sports injuries.

The findings of this study indicated that athletes with muscular and brain injuries (concussed and fainted) did not mention any further encounters with any medical professionals before they returned to sports participation. Yet, proper treatment prior to return-to-sports is regarded is a necessity in re-injury avoidance. 20,26, 42 Annear and colleagues,^2^ investigated the current practices of sports physiotherapists in implementing psychological strategies during athletes' return-to-play rehabilitation to explore their challenges. They revealed that the barriers to implementing psychological strategies included the lack of knowledge and training on this specific strategy. Besides, the participants in our study, indicated that there was a stigma from athletes towards seeking health care support. The findings of this study may suggest gaps in maintenance of athletes' health, rehabilitation and return-to-sports knowledge among the sports resource provders. However, there is a growing body of literature that suggests the need to equip athletes with knowledge that is vital in understanding their bodies, and to become active key stakeholders in return-to-play decisions,^43^, ^44^ However, this might still be subject to some apprehension from coaches and team managers who might perceive some athletes as hurrying to return-to-play.

Our study is among the few 25 that have involved only a trivial proportion of Ugandan national professional athletes participating in 4 of the 48 national sports associations in Uganda. Furthermore, being an observational study, makes it susceptible to biases, confounding and reliability challenges.^45^

## Conclusion

Results from our study should be cautiously interpreted due to its trivial nature. In addition, further research is warranted to establish the level of knowledge and practices among the sports medicine resources providers in Uganda. It is vital to assess the availability and quality of equipment at sports and rehabilitation facilities to assist with adequate care of athletes before and after injuries, and the ability to conduct sports injury surveillance. It is also vital to formulate sports regulations and policies for sports injury prevention and management in Uganda.

## Figures and Tables

**Table 3:** Treatment modalities commonly used during emergency care (on-field care)

Treatment modalities	Abrasion	Concussion	Contusion	Dislocation	Faint	Fractures	Lacerations	Sprain	Strain
Ice	-	-	-	-	-	-	-	14	4
Ice & Bandage	-	1	-	-	-	-	3	6	2
Ice & Massage	-	-	-	-	-	-	-	-	2
Ice & Spray	-	-	-	-	-	-	-	1	-
Ice & Resting	-	-	-	-	-	-	-	-	1
Ice, Massage & Bandage	-	-	-	-	-	-	-	1	-
Painkillers	-	-	-	-	-	-	-	1	-
Immobilization	-	-	-	-	-	-	-	1	-
Bandage only	-	-	-	-	-	-	-	-	1
Stretch exercises	-	-	-	-	-	-	-	1	-
spray & massage	-	-	-	-	-	1	-	1	-
Water	1	-	-	-	-	-	-	-	-
Arm sling	-	-	-	-	-	2	-	-	-
Wound dressing	-	-	-	-	-	-	1	-	-
SCAT3	-	1	-	-	-	-	-	-	-

No service	1	1	1	1	1	-	-	15	9
Total (Number)	2	3	1	1	1	3	4	41	19

**Table 4:** Proportion of injuries managed by conservative approach after the intermediate care phase

	Intermediate care phase		Conservative approach	
**Injury condition**	**Yes**	**No**	**Yes**	**No**
Abrasion	1	1	2	-
Concussions	1	2	3	-
Contusion	-	1	-	-
Dislocations	1	-	1	-
Lacerations	3	1	3	1
Fainting	1	-	1	-
Fractures	2	1	2	1
Sprain	26	15	26	15
Strain	12	7	12	7

**Total**	**47**	**28**	**50**	**24**

## Data sharing statement

The authors can be contacted for data sharing pending approval from the Uganda National Council for Science and Technology.
